# Quantum computing in bioinformatics: a systematic review mapping

**DOI:** 10.1093/bib/bbae391

**Published:** 2024-08-14

**Authors:** Katarzyna Nałęcz-Charkiewicz, Kamil Charkiewicz, Robert M Nowak

**Affiliations:** Artificial Intelligence Division, Institute of Computer Science, Faculty of Electronics and Information Technology, Warsaw University of Technology, Nowowiejska 15/19, 00-665 Warsaw, Poland; Independent Researcher, Warsaw, Poland; Artificial Intelligence Division, Institute of Computer Science, Faculty of Electronics and Information Technology, Warsaw University of Technology, Nowowiejska 15/19, 00-665 Warsaw, Poland

**Keywords:** quantum computing, bioinformatics, mapping review

## Abstract

The field of quantum computing (QC) is expanding, with efforts being made to apply it to areas previously covered by classical algorithms and methods. Bioinformatics is one such domain that is developing in terms of QC. This article offers a broad mapping review of methods and algorithms of QC in bioinformatics, marking the first of its kind. It presents an overview of the domain and aids researchers in identifying further research directions in the early stages of this field of knowledge. The work presented here shows the current state-of-the-art solutions, focuses on general future directions, and highlights the limitations of current methods. The gathered data includes a comprehensive list of identified methods along with descriptions, classifications, and elaborations of their advantages and disadvantages. Results are presented not just in a descriptive table but also in an aggregated and visual format.

## Introduction

### Background

The concept of quantum computing (QC) employs the principles of quantum mechanics, including superposition, entanglement, and quantum tunnelling, to execute computations with greater efficiency compared to classical computers for specific problem sets. ‘In recent years, quantum mechanics has emerged as a tool for analysing biological systems, and the intersection of bioinformatics, biology, and quantum mechanics has led to the emergence of quantum bioinformatics.’ [1]. The origins of QC, can be traced back to the 1980s. Since the early 2000s, there has been a dynamic development in this field, as evidenced by the increasing number of scientific publications referencing ‘quantum computing’. The use of quantum phenomena in specially designed quantum algorithms makes it possible to reduce the computational complexity of tasks that are difficult and computationally expensive to solve using classical methods (quantum supremacy). Of course, it should be emphasised that the use of QC provides a real computational advantage only for selected problems. So far, the number of problems for which the superiority of quantum over classical methods has been scientifically proven remains limited. The field of QC is young and its hardware solutions are immature. QC is a new and exciting area of research that has yet to fully develop—researchers are striving to overcome the challenges of creating more advanced, larger and more reliable hardware, while, at the same time, developing algorithms specifically designed for QC.

The potential applications of QC are vast, ranging from optimisation to quantum cryptography to molecular simulations. One of the fields that could really benefit from the implementation of computational methods based on the quantum paradigm is bioinformatics. Initial attempts have already been made to assess the extent to which QC can deal with complex biological data and computational challenges such as genome sequencing, protein folding and molecular simulation. We aim to find, combine, and organise these studies to understand the big picture and explore the future possibilities that come from both fields working together.

### Motivation

Systematic review mapping is a method of literature analysis that involves structurally examining, classifying, and organising the available scientific literature in a particular subject area. Unlike a traditional systematic review, where the main aim is to analyse existing research, systematic review mapping focuses on identifying the scope and breadth of research within a topic area.

There are several reasons for our focus on the application of QC in bioinformatics. Firstly, the decreasing cost of acquiring biological data (e.g. the decreasing cost of DNA sequencing) and the consequent significant increase in the amount of data has created a need for new methods of analysis. Currently, the bottleneck in bioinformatics is not the data acquisition process, but the computational part. There is therefore a need to further improve existing algorithms and tools, particularly in terms of scalability and efficiency. In addition to attempts to parallelize data processing using GPU computing, dedicated FPGAs, or large computing clusters, one possible way forward is to exploit the QC paradigm.

On the other hand, interest in the quantum paradigm has been growing steadily in both the scientific and business communities. We have just entered a quantum age, comparable in its revolutionary nature to the quantum transistor revolution that began in the late 1940s [2].

The justification for our curiosity about the quantum paradigm and its applications can be furthered by a philosophical analysis. Every computation is, in fact, a quantum computation; classical computation is only a particular subset of it (‘Any classical computation can be done on a quantum computer. Consequently, quantum computation is more general than classical computation. Quantum computations are not a strange way of doing a few special calculations; rather, they are a new way of thinking about computation as a concept. We shouldn’t think of quantum and classical computation as two distinct subjects. Computation is really quantum computation. Classical computations are just special cases of quantum ones.’[2]). One can therefore consider the study of quantum algorithms as such to be ‘justified’, without necessarily—at least not always—trying to demonstrate their possible superiority over the quantum paradigm in specific cases.

In order to gain an overview of the most recent research in QC in bioinformatics and identify key areas of interest, we have reviewed a series of review articles that provide insights into this field. The article [1] describes the combination of QC and bioinformatics in an interesting and systematic way, while also distinguishing quantum biology. However, it does not increase the scope relative to our article. In another paper [3], the advantage of quantum solutions in bioinformatics applications is demonstrated, alongside specific examples. However, this work does not constitute a systematic review mapping. In the article [4], the authors first explain the basic concepts of QC and summarise the most important and computationally challenging bioinformatics problems, focusing on the modelling of biological molecules and genome assembly. Nevertheless, it is not a systematic review mapping. [5] is a review of articles on drug discovery. The authors summarise, but narrow in scope compared to our work. The focus of [6] is on demonstrating the potential of QC in the field of mental health. The author notes that in order to improve the overall understanding of mental illness and make appropriate diagnoses, these activities require computationally intensive analyses of complex data sets. The work broadens the scope of applications by drawing attention to a field that has hitherto been treated in a marginal manner. The [7] focuses in detail on quantum mechanical, protein folding and drug design problems. In [8], the authors search extensively and in depth for areas where quantum solutions would have an advantage. The article is significant for our review because of the diversity of the fields that the authors cover. The potential of quantum computers in the field of chemistry is explored in depth in [9]. The authors highlight the value of a hybrid approach as a means of optimally representing problems on quantum computers. The article by [10] is a systematic review mapping the use of QC in healthcare systems. As such, it is a subset of our set of interests. Lastly, [11] provides a concise overview of QC in the context of the field of nucleic acid research. Despite the novelty of the most recent work, a cursory examination reveals that the majority of the literature does not qualify as systematic review mapping and lacks the broad scope of our study. Currently, no comprehensive study exists that summarises the current state of knowledge on the applicability of QC in bioinformatics through a systematic review mapping. The present work represents the first attempt to systematise in such a way the research done at the intersection of QC and bioinformatics over the last decade.

### Objectives

The primary objective of this paper is to provide a comprehensive overview of the current state of QC in bioinformatics applications. Furthermore, we attempt to answer the question of to what extent the practical application of QC in the field of bioinformatics is limited by the computational power of currently available devices, and possibly identify those areas of bioinformatics where it is already known that the quantum paradigm may have a real advantage over computation performed in the classical model.

For the purposes of this systematic review mapping, the following research questions have been defined:

**Q1:** What is the current state of research on the use of QC in bioinformatics?
**Q2:** What areas are mentioned as potential places where QC can assist in bioinformatics issues?
**Q3:** Which quantum approaches were most often used to solve problems in the field of bioinformatics?
**Q4:** What are the current challenges and obstacles in applying QC to solve bioinformatics problems?
**Q5:** What are the key areas for future research at the intersection of QC and bioinformatics?

The rest of this article is structured as follows: in Section 2, we outline the systematic methodology employed for this review, detailing research questions, inclusion criteria, and the strategic search methodology. Section 3 captures emerging trends at the intersection of QC and bioinformatics. Section 4 presents and analyses the key findings, trends, and connections from the literature review. Finally, Section 5 synthesises our observations and Section 6 concludes the paper with a discussion of future work.

## Methodology

This section describes the selection process for the literature items analysed in this systematic review mapping.

### Inclusion criteria

For the purpose of conducting the systematic review mapping, we formulated the following criteria:

- **Importance of the topic:** While our primary focus lies on studies that deal with the application of an algorithm or a set of quantum or hybrid algorithms to solve a certain bioinformatics problem, we also took into consideration some works that are quantum-inspired. These quantum-inspired works were selected based on the terminology used by their authors to describe the algorithms they employed, which was often found in the title, keywords, or throughout the body of the paper. We made a point to highlight such articles (see Table 1) to assess the extent of research in this area.
- **Language:** We decided to include only articles written in English, mainly because it is undoubtedly the language that is the standard for scientific communication in the fields of computer science, bioinformatics, and QC. This assumption also makes it possible to repeat our research at a later date.
- **Type of publication:** In order to ensure the appropriate quality of the publications we analyse (journal articles and conference proceedings), we have decided to focus only on items that have passed the review process (mostly peer-reviewed journals/conference proceedings).
- **Date of publication:** Bioinformatics and quantum information science are dynamically developing fields of science. For this reason, in order to avoid the analysis of obsolete items, we decided to include in our analysis publications from the beginning of 2012.
- **Accessibility:** To enable possible repetition or re-evaluation of our research, one of the inclusion criteria used was the availability of the analysed items, either through open access or institutional subscriptions.
- **Study design:** In the final set of articles, we decided to include only those that dealt with the original (whether theoretical or practical) application of a quantum/hybrid algorithm to a bioinformatics problem. These could be mathematical analyses, proof of concept publications, or computational experiments. In the detailed analysis, we did not take into account opinion articles, review articles, or those articles that only briefly mentioned the use of QC in the field of bioinformatics (those for which bioinformatics application was not the main goal).

**Table 1:** Overview of trends at the intersection of QC and bioinformatics: categorised literature references. Publications [12–19] belonging to the initial set (‘seeds’; see Section 2), are underlined. Quantum-inspired works [16, 17, 19–23] are highlighted with a light green background

**Categories**	**Works**
Quantum Advancements in Phylogenetics	[24–27]
Quantum Approaches in Sequence Analysis	[20, 28–34] [13][14][12, 15]
Quantum Computing in Graph Analysis and Kernels	[35–41]
Security and Privacy with Quantum Approaches	[26, 42–45]
Quantum Approaches in Biomolecular Structure Prediction and Modeling	[16, 33, 46–61]
Biologically Inspired Quantum Computing	[35, 42, 46, 62]
Quantum Solutions in Data Analysis and Clustering	[33] [21, 63–70]
Quantum Optimizations in Bio-computing	[22, 29, 33, 71, 72] [16–19][20]
Quantum Potential in AI and Advanced Modeling	[23, 51, 60, 73–76]
Additional Applications and Considerations	[77] [78, 79]

### Search strategy

In selecting a literature search strategy, we followed the guidelines outlined in [80]. These guidelines provide a detailed description of the process for conducting a systematic literature review using the ‘snowballing’ method of replicating research in the field of software engineering. In summary, the process involves defining a starting set of literature items and then analysing the references of each item (backward snowballing) as well as the citation sites (forward snowballing). Ideally, the process of adding new candidates should end when no new items can be added to the collection. This happens when items are duplicates or do not meet the basic inclusion criteria, such as language or year of publication. At each stage of the snowballing method, when potentially new items were added to the pool of articles, duplicates were identified and removed using the Digital Object Identifier and the Entry Identifier (Scopus). The relevance of each item (in terms of its potential application of quantum or quantum-inspired algorithms in bioinformatics or related areas) was evaluated based on an analysis of its title, abstract, and, where necessary, the full text. The exact steps of the selection process are described in Section 2.

Snowballing has an important advantage in that it allows the identification of relevant studies that may not have been included in the original search strategy, e.g. when relying only on database searches. It also allows the identification of key authors and experts in the field, as well as relevant conferences and journals, contributing to the diversity of perspectives and credibility of the systematic review. Including studies that may not have been published in high-impact journals helps to reduce publication bias.

In developing the search strategy, our main aim—again—was to carry out a systematic review mapping. It is important to note that our aim was not to identify all relevant articles in the area of interest, as this would have been impossible. Instead, we focused on identifying a sample of elements from the research literature, creating a ‘seed’. Seeds are an initial set of primary studies that are crucial to the literature review process. They represent the initial pool of studies from which further relevant literature is identified. The purpose of seeds is to define the scope of the review and to ensure that the search is comprehensive and systematic. They are essential for maintaining the transparency and reproducibility of the review process and provide a clear starting point for identifying relevant literature.

In [81], the authors suggest that the best results for creating a literature collection are achieved through a combination of methods, such as searching databases and analysing references and citation sites. This approach was also used in our literature search.

Due to limited resources, our systematic review mapping only analysed a well-selected subset of literature items related to the given issue, using backward and forward snowballing. The stopping criterion was not the inability to add new articles to the collection under consideration. In the context of backward snowballing, we analysed only those items that met the basic inclusion criteria and had more than two citations. For forward snowballing, the stop criterion was based on achieving ‘one level of inclusion’. We analysed the citations of the items and evaluated their potential for further consideration in our analysis based on their year of publication, title, and abstract.

Finally, at the end of this section, we would like to refer to the question of the assessment of quality. In this work, which is a mapping systematic review, it was decided not to assess the quality of the items reviewed for several important reasons. As noted in [82], in the case of mapping studies, quality assessment is *‘not essential. Also complicated by the inclusive nature of the search which can include theoretical studies as well as empirical studies of all types making the quality evaluation of primary studies complicated’*. Aware that this decision carries the potential risk of including articles of varying quality in our study, we strictly adhered to the inclusion of only peer-reviewed papers. Additionally, it is challenging to assess the quality of research in QC (QC) applied to bioinformatics due to the nascent nature of the field. At this stage, objective criteria for quality assessment are lacking, and we chose not to impose our own criteria.

### Selection process

**Initial/test set**

In designing the queries for the Scopus database described in the following subsections, an initial/test set was used (see underlined items in Table 1). This set consisted of articles that were deemed relevant to the search results of our queries and was used to verify the relevance of our queries.

**First iteration**

Scopus was chosen as the database because of its comprehensive coverage of scientific literature across a range of disciplines, extensive indexing of prestigious journals, advanced citation analysis capabilities, and the presence of an API [83] that allows partial automation of the article selection process.

1. Making two queries to the Scopus database:
  - **Q1:** KEY ( quantum ) AND ( KEY ( bioinformatics ) OR KEY ( computational AND biology ) ) AND LANGUAGE ( english ) AND PUBYEAR > 2012
  - **Q2:** TITLE ( quantum ) AND ( TITLE-ABS-KEY ( ( computational AND biology ) ) OR TITLE-ABS-KEY ( bioinformatics ) ) AND LANGUAGE ( english ) AND PUBYEAR > 2012
2.   Query Q2 was added after verifying the results obtained with Q1. It was discovered that the results obtained for Q1 did not include the paper [20] from the initial/test dataset.
3. Removing duplicates.
4. Analysing the abstract titles and selecting articles for the second iteration.

**Second iteration (backward snowballing)**

1. Analysis of the references of articles that qualified for the second iteration. Only references that appeared at least twice were selected.
2. Analysis of the titles and abstracts of articles resulting from backward snowballing. The selected articles were included in the final set.

**Third iteration (forward snowballing)**

1. For all articles that qualify for further processing, a citation analysis was conducted. The titles and abstracts of the citing items were considered to decide whether they should be included in the final collection.

**Final processing**

1. Screening—a quick review of the content of all articles initially classified in the final collection and discarding those that do not fit thematically.
2. Full text overview—analysis of the publication based on a reading of the full text.

### Data extraction and analysis

To ensure a consistent overview of the selected articles, we created a comprehensive data extraction form. This form enabled the methodical collection of relevant information and enabled us to analyse the content in a structured and consistent manner.

1. **EID:** Electronic Identifier; unique alphanumeric identifier (from Scopus database) for each document.
2. **Year:** Publication year of the document.
3. **Affiliation countries:** Countries of the affiliations of the authors.
4. **Venue type:** Type of publication venue (e.g., journal, conference).
5. **Author keywords:** Keywords provided by the article’s authors.
6. **Type of the article:** Categorised as Empirical, Theoretical, or Both.
7. **QC Approach:** Specific approach or technique from quantum computing applied in the article (Quantum algorithms, Hybrid quantum-classical algorithms, Quantum machine learning, Quantum annealing, Quantum simulation, Topological quantum computing, Quantum communication and cryptography).
8. **Area of bioinformatics:** Specific domain within bioinformatics that the study addresses (Genomics and Transcriptomics, Metabolomics, Phylogenetics, Systems Biology, Structural Bioinformatics, Machine Learning and Data Mining in Bioinformatics, Functional Genomics, Epigenomics).
9. **Major contribution of the article:** Primary contributions like Process, Methodology, Models, Tools, among others.
10. **Research type:** The nature of research methodology used.
11. **Data:** Type or description of the data used in the study.
12. **Aims and objectives:** Main goals and targets of the article.
13. **Findings and conclusions:** Principal outcomes and conclusions drawn by the authors.

The following fields in the data extraction form were extracted automatically using an export from the Scopus database: EID, Year, Affiliation countries, Venue type, and Author keywords.

## Trends in QC for bioinformatics

An Analysis of the 68 articles has allowed us to identify some trends among researchers working on QC in the context of bioinformatics. This section is an attempt to organise and systematise them. It is important to note that the identified groups have fluid boundaries, and individual articles may fit into more than one trend. In certain cases, this type of work has been included more than once, while in others we have chosen to assign it to a trend that we feel best reflects its nature.

Trends have been distinguished multidimensionally, both in terms of the type of bioinformatics problem solved (e.g. DNA sequence assembly, protein folding, sequence alignment), the QC model used, the category of computational task (e.g. optimisation, image segmentation, or data security), and the direction of the flow of scientific inspiration (starting with a biological problem and seeking a solution in the QC domain, or vice versa, biological inspiration finding an application in the QC domain). It should be noted that the trends highlighted do not cover all possible dimensions and divisions in this complex and multifaceted field. Rather, they represent our arbitrary selection of those aspects of the field analysed that seemed to us to be the most relevant and, in our view, the most reflective.

One of the things we wanted to look out for in the papers we selected was whether there was an obvious tendency to transfer algorithms/attempts to solve bioinformatics problems from classical to quantum methods. In other words, are attempts being made to approach a particular bioinformatics problem immediately in a ’quantum’ way? Our analysis shows that such a trend is continuing, i.e. at present the thinking of bioinformaticians trying to use QC to solve bioinformatics problems is still moving from a classical to a quantum approach. It seems that the reasons for this state of affairs can be traced back to the immaturity of QC—it is still a novel approach that is not very well established in the bioinformatics community. Additionally, the limitations of available hardware play a significant role. As a result, classical solutions (i.e. computations on classical computers) are still the first to be considered when faced with a bioinformatics problem/challenge. On the other hand, it can be seen that researchers are exploring the potential of QC to address bioinformatics challenges, and that the number of cases where quantum solutions to specific bioinformatics challenges are directly explored is increasing.

One fairly clear observation that comes to mind is the early stage of development of the field linking QC and bioinformatics. This observation is not surprising, given that the world of QC is itself at an early stage, not only in terms of the maturity of the hardware available, but also in terms of algorithm development. Moreover, not only is the number of dedicated quantum algorithms relatively small compared to other more mature and well-established areas of computing, but there is still insufficient knowledge about the extent to which QC could help solve complex problems. It can be stated objectively that we are still in the experimental phase, still searching for those potential applications of QC in bioinformatics where the use of QC will result in a significant reduction in the computational complexity of the problem under consideration—achieving quantum supremacy. Among the papers we have reviewed, only a limited number have clearly demonstrated the advantage of utilising QC over classical computing.

Speaking of quantum supremacy, it is impossible not to mention Grover’s algorithm for unstructured databases [84], which, along with Shor’s algorithm for factoring large numbers [85], are the two most famous examples of algorithms that have been shown to achieve quantum supremacy. The first algorithm mentioned, by Grover, also appears in studies at the intersection of quantum computing and bioinformatics. As databases of genomic data continue to grow, there is interest in using Grover’s algorithm to speed up searches in these databases. In the analysed set of articles, we identified several instances where the described algorithms rely on Grover’s algorithm. These include [12, 28–30, 35, 36, 46–49, 63], and [31].

Another important observation, upon analyzing the set of articles selected for this systematic review mapping, is the limited availability of described ready-made tools or frameworks. It is worth noting that there are very few dedicated quantum tools being developed for bioinformatics purposes at the present time, which is understandable considering the early stage of the field’s development.

If we look at the merging of the fields of bioinformatics and QC, although the former initially acted as a ‘consumer’ of quantum methods, over time we could also see the reverse inspiration. This is evidenced by three items in our compilation, namely [42, 62], and [46].

The trends outlined in Table 1 illustrate the role of QC in bioinformatics and the wide range of potential applications—from biological sequence analysis, data analysis (feature selection, segmentation of image data), protein folding, to ensuring the security and privacy of sensitive bioinformatics data using advances in quantum cryptography.

## Results

**Figure 1:**
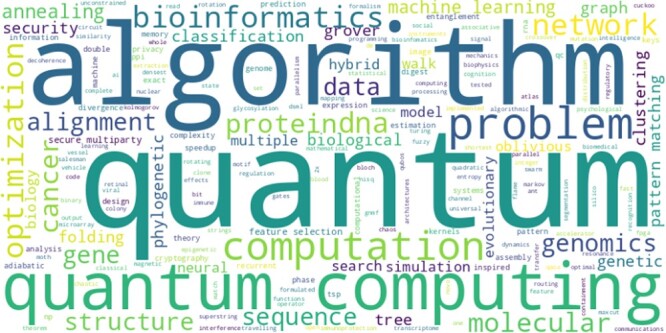
The word cloud above shows the unified and cleaned Author keywords taken from the Scopus database for the papers in the final set of publications. The processed keywords were used to create the word cloud after being tokenized, lowercased, and stripped of stop words and punctuation. The size of each word corresponds to its frequency.

When discussing the results of our systematic review, we will begin with a broad overview of the themes present in the analysed collection of articles. The word cloud in Figure 1 can give us such an idea, allowing a quick understanding of the main research themes in each field.

**Figure 2:**
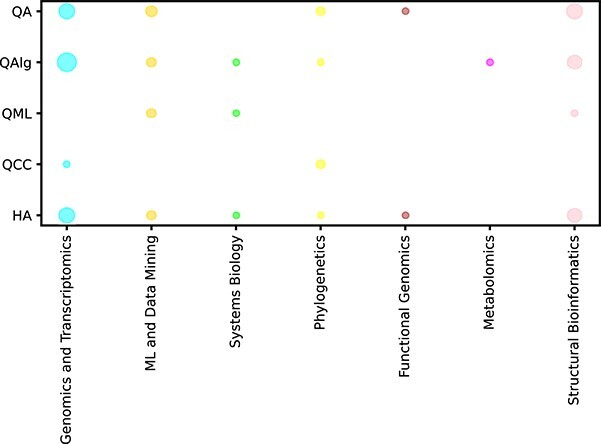
The bubble plot above illustrates the number of studies for different QC approaches in bioinformatics. Each bubble represents a combination of a bioinformatics problem and a QC approach, with the size of the bubble indicating the number of studies conducted on that particular combination. QA: Quantum Annealing; QAlg: Quantum Algorithms; QML: Quantum Machine Learning; QCC: Quantum Communication and Cryptography; HA: Hybrid Algorithms. As shown, the main research interests consist of Genomics and Transcriptomics, ML and Data Mining or Structural Bioinformatics (in the Bioinformatics domain), and General Quantum Algorithms, Hybrid Algorithms, and QA (in the Quantum domain). The focus on the general type of applications (in contrast to specific and more sophisticated topics) is a common phenomenon in the early phase of new solution domain adoption. Some of the fields (Functional Genomics, Metabolomics) have very small representation and still are not a subject of research interest.

Figure 2 attempts to identify the methods used to solve different areas in bioinformatics. The scheme only includes works that can be clearly assigned to specific categories. Publications that were labelled as Other in either the quantum approach or bioinformatics field categories were excluded.

There are two types of working quantum devices: devices based on quantum logic gates (currently IBM has announced a device containing 1000 qubits (A qubit (quantum bit) is *‘the basic unit of information in a quantum computer’* [86].)), and adiabatic computers (quantum annealers) that use quantum operations to find the optimum (currently D-Wave is writing about a version containing 5000 qubits). To address **Q1**, **Q2**, and **Q3**, we decided to depict QC applications based in the following areas: Quantum Potential in AI and Advanced Modeling; Quantum Approaches in Biomolecular Structure Prediction and Modeling; Biologically Inspired Quantum Computing; Security and Privacy with Quantum Approaches; Quantum Advancements in Phylogenetics; Quantum Optimizations in Bio-computing; and Quantum Computing in Graph Analysis and Kernels. Our findings are in section 4.

To address **Q4** and **Q5**, we list the currently the most popular research areas in bioinformatics that correspond to Communities of Special Interest (COSI) within the International Society for Computational Biology (ISCB). COSIs are self-organised communities facilitating year-round interaction and collaboration among members sharing research interests in computational biology (A detailed explanation of COSIs can be found on the ISCB: https://www.iscb.org/ismb2018general/iscb-cosi.). The areas include: Machine Learning in Computational and Systems Biology; High-throughput Sequencing; Regulatory and Systems Genomics; Translational Medical Informatics; Bio-Ontologies; Evolution and Comparative Genomics; Structural Bioinformatics and Computational Biophysics; Network Biology; Computational RNA Biology; Variant Interpretation; Biological Data Visualization; Computational Mass Spectrometry; Protein Function Annotation; Microbiome; Critical Assessment of Massive Data Analysis; Computational Biology Education; General Computational Biology; Text Mining; and Computational Modeling of Biological Systems. We analyse how many QC papers address these problems. Such results are presented in Section 4.

Several articles lack experimental data, with 19 having no data due to their theoretical nature. Additionally, 14 articles only provide a toy example due to the early phase of research on the topic. The remaining papers were prepared using larger sets of real, synthetic, or random data. This information about datasets, together with info about type of hardware used, the nature of the research itself (theoretical and/or empirical) and whether the dataset represents real data or a toy model, are summarised in Table 2. The review indicates that while the presented research is promising, it should be extended and reproduced on larger datasets to demonstrate its real usefulness.

**Table 2:** This paper presents a summary of the reviewed articles, categorising them according to the type of hardware used, the nature of the research (empirical or theoretical), and whether the dataset represents real data or a toy model. Articles conducted on real hardware and analysing real datasets are highlighted, as they appear to be more relevant to real-world applications.

Works	Type		Hardware				Dataset		
	Theoretical	Empirical	No	Annealer	Simulator	Other	No or N/A	Real	Toy
[24, 41]	✓	✓		✓					✓
[12, 17, 18, 22, 25, 28, 37–39, 51, 65–67, 69, 72, 74–76]	✓	✓			✓			✓	
[30, 31, 34, 36, 47, 56, 63, 73, 77]	✓		✓				✓		
[13, 33]	✓	✓		✓	✓				✓
[23, 53]		✓		✓				✓	
[26, 43]	✓	✓	✓				✓		
[78]		✓		✓	✓			✓	
[52, 59]	✓	✓		✓				✓	
[21]		✓			✓			✓	
[15, 70]	✓	✓		✓				✓	✓
[27]	✓	✓		✓		✓		✓	✓
[16, 19, 29, 32, 45, 48, 60, 62]	✓	✓			✓				✓
[40]	✓	✓			✓			✓	✓
[35]	✓		✓						✓
[64]	✓	✓	✓					✓	
[54, 68]	✓	✓		✓	✓			✓	
[49, 58]	✓	✓			✓	✓			✓
[55, 57]	✓		✓	✓			✓		
[61]	✓				✓	✓			✓
[50]	✓	✓				✓		✓	
[71, 79]	✓	✓			✓		✓		
[20]	✓	✓		✓	✓			✓	✓
[14]	✓				✓		✓		
[44]		✓				✓	✓		
[42]	✓	✓				✓	✓		
[46]	✓	✓				✓			✓

### Applications of QC in bioinformatics—overview

#### Quantum potential in AI and advanced modelling

We will start our analysis of QC applications in bioinformatics by discussing its potential in artificial intelligence (AI) and advanced modelling.

The study conducted by [78] focused on evaluating the capabilities of Quantum Machine Learning (QML), specifically using QA, applied to the problem of classification and ranking of transcription factor binding affinities for a specific computational biology problem. QA showed a slight advantage in classification performance and almost equal ranking performance compared to classical approaches.

The potential of Ising-type algorithms in biomedical research, especially in multi-omics classification of human cancer data, is also highlighted in [23]. Comparing these algorithms with standard machine learning approaches, the authors highlight the high accuracy of QC-based methods, especially for small input datasets and limited training data.

The paper [74] demonstrates the effectiveness of QML in improving the accuracy of force fields in molecular simulations, specifically when stretching DNA base pairs in explicit solvent. This work is a significant advancement in the field of molecular dynamics simulations and computational chemistry.

[75] propose a new approach to cancer transcriptomics analysis using the quantum k-means clustering algorithm, achieving high accuracy in classifying different cancer types.

The authors of [76] utilise a quantum ant colony optimisation algorithm to enhance the performance of a quantum-inspired neural network. The objective is to prevent the network from becoming trapped in suboptimal solutions. The presented solution is used for predicting protein-protein interactions (PPI) and achieves better results than support vector machine (SVM) and artificial neural networks.

In turn, the paper [51] focuses on the problem of protein structure prediction by combining QC with genetic evaluation. The quantum genetic algorithm presented here demonstrates lower memory complexity and faster running time than classical alternatives.

The authors of [60] refer to the Radical Pair Mechanism, which modulates the kinetics of chemical reactions by influencing spin dynamics, and use a toy-sized example to describe a hybrid QML algorithm for simulating spin systems.

#### Quantum approaches in biomolecular structure prediction and modelling

The paper [51] is on the borderline between two distinct areas of research: one pertaining to quality control (QC) and AI in the context of bioinformatics, and another encompassing a multitude of works addressing biomolecular structure prediction and modelling. An interesting paper in this area is [77], which deals with the use of quantum theory and measurement theory to model information processes in biosystems. The authors model cognitive effects and gene regulation in Escherichia coli bacteria. The aim is to develop a mathematical framework that can better describe the behaviour of biological systems, which, as the authors point out, differ significantly from classical mechanical systems.

The paper [50] presents a hybrid algorithm for computer-based modelling of protein binding sites. The algorithm is specifically designed to identify potential binding signals of the SP-A. Additionally, the paper introduces a model simplification method called the QAOA-MaxCut protein pruning tool, which is based on the quantum approximate optimization algorithm (QAOA) and is utilised in this context.

The algorithm described by [48] offers a method for predicting the 3D structures of proteins that efficiently captures the essential hydrophobic-hydrophilic interactions by translating sequences onto a body-centred cubic lattice. The potential of QC is demonstrated here by the use of Grover’s algorithm in the critical step of searching for candidate confirmations. The significantly lower computational complexity of the method compared to classical alternatives is demonstrated.

The field of protein structure prediction was also explored in [55], where the aim was to evaluate the potential of adiabatic quantum computation in the field of protein structure prediction. The potential of the quantum approach was demonstrated, while emphasising the need to optimise the quantum models used to improve their accuracy.

The article [48] proposes an algorithm for predicting protein 3D structures that efficiently captures hydrophobic-hydrophilic interactions by translating sequences onto a body-centred cubic lattice. The potential of QC is illustrated through the application of Grover’s algorithm in the critical step of searching for candidate confirmations. The method also demonstrates significantly lower computational complexity compared to classical alternatives.

[52] is another paper dealing with the problem of predicting the structure of biological sequences—here the focus is on predicting the secondary structure of RNA, where the task is formulated in the form of a binary quadratic model (BQM). QA was used in the solution, proving the effectiveness of the proposed method, especially on a set containing known structures with pseudo-nodes.

QA has also been applied to the protein folding problem in [53]. The authors address the scalability concerns associated with this issue, which is formulated as an optimisation task for a quantum annealing (QA). By comparing this method with classical approaches, they prove that it is possible to outperform them with an algorithm based on quantum computers. At the same time, they point out that the magnitude of the speedup is small and may not be practically significant.

The issue of protein design is also addressed by [54], who present the XNet tool designed to improve the performance of protein modelling tools on quantum computers.

PPI was the focus of the paper [56], which described a new method based on quantum walks to identify structurally similar subnets in PPI networks.

The paper [16] focused on the protein folding problem. They proposed a quantum-inspired evolutionary algorithm that uses a variable angle-distance rotation strategy. The algorithm was shown to be able to find optimal or near-optimal energy structures and demonstrated superior performance and feasibility compared to other evolutionary algorithms.

The [58] also focused on protein folding and presented an algorithm that combines quantum walks and deep learning together with the Metropolis algorithm. The method presented has the potential to offer polynomial speedup compared to classical methods.

Protein folding was also the focus of [61], where a new resource-efficient method was proposed. The algorithm used a Hamiltonian model and a Variational Quantum Algorithm to simulate the folding of polymer chains on a lattice, with an emphasis on efficiency, achieved in part by incorporating physico-chemical properties of proteins into the model.

In turn, [59] addressed the issue of RNA folding and presented a quadratic unconstrained binary optimization (QUBO) model for this task, optimised by variational hybrid QA and suitable for both quantum walk and quantum gate based computers. Its effectiveness in structural bioinformatics applications was confirmed.

In [49], a quantum circuit was designed to identify nucleotides in single-molecule sequencing data, and a hybrid quantum-classical algorithm was presented to solve this problem. The authors point to the potential ofQC, in particular the Grover algorithm, in personalised medicine, drug discovery or diagnostics by enabling fast analysis of large data sets with combinatorial problems.

The aim of [57] was to reduce the task of finding the most probable transition paths in thermally activated conformational reactions to a shortest path problem and to map it to the Ising model. A QA approach was used. The developed method does not require lattice discretisation. The method was designed to be applicable to realistic molecular models.

The potential of QC, in particular the Grover algorithm, in the PPI problem was discussed in [47]. The algorithm was compared with classical ML algorithms and showed improved prediction accuracy.

In [46], a biomolecular algorithm was proposed as an extension of Grover’s algorithm. This method utilises a QC paradigm to infer the value of individual bits of a solution state in an unordered database. The authors demonstrate the algorithm’s efficiency by solving a clique problem.

The paper [71] describes a quantum algorithm based on quantum phase estimation for similarity measures for molecules, and shows the possibility of achieving exponential speedups with this method compared to classical algorithms. The algorithm can be used, for example, in the context of chemical compounds, drug discovery, and molecular analysis.

#### Biologically inspired QC

The paper by [35] describes a quantum algorithm for implementing Boolean circuits derived from the DNA-based algorithm for solving the vertex cover problem in finite dimensional Herbert space, and presents the results of a nuclear magnetic resonance (NMR) experiment testing the proposed theory.

The work of [42] is another example of QC taking inspiration from the field of bioinformatics, a reversal of the typical direction of the relationship between these fields. Namely, the concept of DNA coding was applied to a quantum cryptographic algorithm for secure key exchange, where the unique properties of DNA sequences were used to create a highly secure random transformation for additional protection of augmented keys.

The article [62] provides further evidence that the field of QC can be inspired by bioinformatics research. The authors focused on exploring the potential of biologically motivated quantum neural network (QNN), with the goal of creating machine intelligence that more closely resembles biological intelligence.

#### Security and privacy with quantum approaches

In continuation of the theme of quantum-inspired approaches intersecting with both biologically inspired computing and security, the paper [19] presents another quantum-inspired genetic algorithm, QIGA, which solves the double digest problem (DDP) and has practical applications in physical mapping of DNA. Its effectiveness and efficiency are demonstrated by simulation results on a classical computer. An extension of this work is [45], where the authors design a framework for solving the DDP, with special emphasis on the security of DDP data.

A proof-of-concept system for private detection of composite signals in genomes and proteins using quantum technologies, based on the generation and distribution of quantum oblivious keys, was presented in [43]. The aim was to enable a variety of entities to validate sensitive data on genomes and proteins without the risk of disclosure to third parties.

A similar goal guided the research summarised in [26], where the authors presented a system dedicated to the secure computation of phylogenetic trees of the SARS-CoV-2 genome. By secure, they mean a method that allows the construction of phylogenetic trees based on data collected by different entities, without the need to disclose the data in question to each other. The quantum part of the system is responsible for the security and privacy of this data.

The authors of [44] developed a novel system for secure secondary utilisation of genomic data using quantum-secure cloud technology, allowing secure multi-user access to analyse large genomic data.

#### Quantum advancements in phylogenetics

The work [25] provides a seamless transition from data privacy protection to phylogenetics. The main motivation of the authors was to ensure privacy and protection in the data analysis process. The authors developed a hybrid system using QC for the privacy part of the phylogenetic tree computation process. The superiority of the hybrid (quantum-classical) application in terms of information security assurance and efficiency over a purely classical approach was demonstrated.

The paper [24] contains a new formulation of the tree containment problem (belonging to the class of NP-complete problems) in the form of QUBO. The authors experimentally confirm the effectiveness of this approach for small data sets, showing that the number of physical qubits does not grow exponentially with the size of the input data. This allows us to hope for the practical application of the developed method with the development of available quantum processing unit architectures, including a larger number and connectivity of qubits.

A unique work in the collection we selected for this study is the [27], due to its use of a quantum-inspired computer, the Fujitsu Digital Annealer. In this work, it was used to construct phylogenetic trees, achieving similar results (in terms of optimality) to existing methods, while reducing processing time.

#### Quantum optimizations in bio-computing

We can also find quantum-inspired work (see 3) in the area of optimization. The paper [22] is another example of quantum-inspired work, proposing optimisation heuristics for the multiple sequence alignment (MSA) problem in bioinformatics, with promising results, for example, in cases where no prior knowledge of the relationship between sequences is known and fast (and possibly accurate) alignment is required.

In [18], a new hybrid swarm intelligence algorithm for gene selection based on QC and moth flame optimisation was described in applications to the problem of gene selection in microarray data, which had better performance of classification accuracy than existing classical competing approaches. Quantum-inspired operations were used to update the position of the moths in search space, which allowed more efficient exploration of the search space.

A new quantum evolutionary algorithm based on the Bloch quantum chaos algorithm for constructing DNA code sets that satisfy certain distance constraints is described in [79]. The authors point to its potential applications, including information encryption and the design of new drugs and therapies. This is another example of QC drawing on bioinformatics.

A new method based on QC for reverse engineering gene regulatory network (GRN) from time-series generic expression data is proposed in [72]. The method described is shown to significantly reduce computation time while maintaining the required level of accuracy.

[17] is another work that has aimed to apply a QC paradigm to the MSA problem. A new hybrid method based on global/local search alignment and the quantum cuckoo search algorithm, which is used to explore the search space and find the optimal solution, is presented. It is shown that the proposed method improves the results by reducing the population size and increasing the diversity due to quantum encoding.

#### Quantum approaches in sequence analysis

Moving on to another important area of study, we will now examine sequence analysis. The work of [20] applied quantum and quantum-inspired annealing to the task of genome assembly. The goal was to investigate the potential of QA in this area. Graph partitioning was used to break down the problem into smaller sub-problems. The study concluded that the proposed method shows promise, but further research is required.

Also in [13], the problem of DNA sequence reconstruction using a hybrid (quantum-classical) and QA algorithm, as well as gate-gated QC, was approached, leading to similar conclusions.

A similar goal was pursued by the authors of [15], where a method based on the formulation of de novo assembly as a QUBO problem was also described and solved again using QA, by performing experiments on fragments of real genomes of organisms.

The paper of [32] uses a quantum algorithm based on the quantum amplitude amplification technique to amplify the amplitude of the projected solution state for the DNA read alignment task. The authors use a gate model and analyse in detail the proposed solutions in terms of complexity, considering both the number of qubits and gates involved. They highlight the potential of the quantum approach to improve the accuracy and efficiency of read alignment by reducing the number of false positives and false negatives, as well as possible applications in areas such as gene ontologies or genome-wide association studies.

In [28], QC was applied to one of the fundamental and key problems in bioinformatics, namely exact multiple pattern matching in biological sequences. Using quantum counting algorithms and shared quantum memory, the algorithms discussed here address the challenges of processing large biological datasets, allowing more efficient exploration of the search space.

Also, [29] recognises the potential of QC for parallelizing computation and efficiently searching the solution space in the exact pattern matching problem, especially in the context of biological sequences and large text databases from biological and biomedical experiments.

QC has been applied in [31]’s work to the Short Super String problem, which is crucial for DNA sequence assembly, among other applications. The algorithm was based on Grover’s algorithm.

The work of [34] is of a theoretical nature. It is shown, based on theoretical analysis, that the proposed algorithm can be used to discover a DNA motif model using the adiabatic quantum algorithm.

The [30] focuses on the use of a quantum multi-pattern recognition algorithm, which is an extension of Grover’s algorithm, combined with dot matrix approach to improve sequence alignment.

A new quantum algorithm for short-read alignment that is feasible for use in genomics and transcriptomics, and that achieves improvements in speed and accuracy over existing aligners, is described in [14].

The aim of [12] was to explore the utility of QC in sequence matching (especially complex RNA structures) in genomics. A new algorithm based on quantum pattern recognition, quantum Hamming distance and Grover’s search algorithm was presented. The authors demonstrated the possibility of achieving quadratic speedups with the proposed method.

#### QC in graph analysis and kernels

Three papers [37–39], partly linked by a common group of co-authors, are devoted to the problem of graph analysis and classification. By computing the quantum Jensen-Shannon divergence (treated as a graph kernel), the dissimilarity between graphs is measured. In each of these papers, the authors emphasise the importance of the described methods in bioinformatics (as one of the fields, besides e.g. computer vision, where they can find potential application), perform experiments on bioinformatics datasets and demonstrate the superiority of the proposed method over classical alternatives.

The authors [40] also propose a quantum graph kernel and emphasise the particular value of using quantum superposition, and hence lower computational complexity compared to classical kernel function computation, in the field of bioinformatics, where graph classification tasks are common. The proposed quantum graph kernel is shown to achieve higher accuracy in the classification task compared to classical methods.

The focus of [36] is on the multiple network alignment problem—computing the topological similarity of nodes in a given set of graphs, a problem with wide applications including chemoinformatics and bioinformatics (e.g. computing the pairwise similarity of molecules). A quantum phase estimation algorithm was used to solve it. The possibility of significant (exponential) speed-up over classical methods was also demonstrated.

In the paper [41], the densest k-subgraph problem was formulated in QUBO and Integer Linear Programming form and QA was used to solve it. Experiments were carried out on bipartite graphs because of their particular importance in computational biology. The study demonstrated how QA can solve computational biology problems represented in graph form.

#### Quantum solutions in data analysis and clustering

Now, we will explore how quantum methods can be applied to data analysis and clustering in the context of bioinformatics.

The article [64] describes a method for efficient clustering of gene expression data that combines graph-regularised non-negative matrix factorisation with quantum clustering, achieving better results than the three classical methods.

The authors of [65] propose a new machine learning method based on quantum mechanics and fuzzy logic for the clustering task and demonstrate its superiority over several currently used clustering algorithms in terms of clustering accuracy and computational efficiency. They highlight its potential applications in, among others, economics, bioinformatics and Big Data. The aim of the work [66] was to develop a new method for vessel segmentation in retinal images using a quantum mechanics-based algorithm. The results were better than many state-of-the-art methods in terms of accuracy. This is an example of an algorithm inspired by quantum mechanics and designed to run on classical computers.

The problem of cancer data classification was addressed in [21], where a method based on a deep learning approach was used in the feature selection part, achieving high accuracy in the classification process. Inspiration from quantum mechanics includes both the method of representing data and the set of operations on it.

[67] is a theoretical paper developing methods for estimating algorithmic information using QC, with applications in genomics such as DNA sequence reconstruction, pattern matching, recognition, and generation.

In [68], both types of quantum computers (QA and gate-based) were used to investigate their usefulness in solving the problem of messenger ribonucleic acid codon optimization. It was shown that the D-Wave Systems Advantage 1.1 was able to solve this task up to approximately 180 codons.

The authors of [69] used Quantum Convolutional Neural Network to classify early stage ischaemic heart disease diagnosis, with promising results compared to classical Optimised Convolutional Neural Network and Fully Connected Neural Network methods.

In [70], the possibilities of kernel-based SVMs on D-Wave QA were investigated. An SVM formulation in the form of QUBO was proposed and SVMs implemented on QA were compared with traditional ones. It was shown that with a method based on QC it is possible to obtain a collection of classifiers that often outperform a single classifier obtained with classical SVMs.

### QC applications in key bioinformatics areas

**Figure 3:**
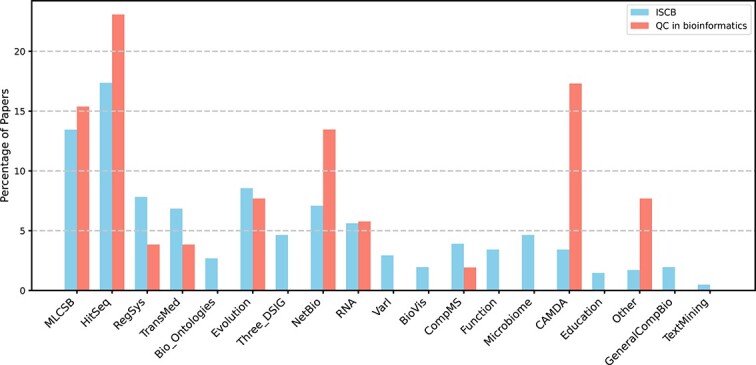
The percentage distribution of papers across different COSIs at ISMB conferences from 2017 to 2023 and COSI assignments in this study’s dataset.

To get a better idea of how the interest of researchers working at the intersection of bioinformatics and QC is distributed, we decided to make the following comparison. We collected statistics on the classification of conference publications for the individual COSI at ISMB conferences from 2017 to 2023. The data focus specifically on the years since 2017, as previous proceedings used less defined ‘areas of interest’, making it difficult to consistently categorise papers across different tracks or topics. We then mapped the articles from the collection analysed in this paper to the aforementioned COSIs. The percentage of each COSI for both sets of scientific articles is shown in Figure 3.

We can see that QC is applied mostly in terms of fields requiring processing massive data sets or with CPU demand like machine learning, sequence processing, or network biology. Still, there are a lot of fields not covered at all. Additionally, the main interest is primarily visible in a few domains, such as sequencing, machine learning, and general computational biology.

## Discussion

The research questions formulated in Section 1 have been addressed in Section 3 and Section 4, where the current state of research on QC applications in bioinformatics (Q1) is comprehensively outlined. We also identified areas within bioinformatics where it is possible to benefit from quantum supremacy either now or in the near future (Q2), as well as the most commonly used quantum approaches in this context (Q3; see in particular Figure 2). Regarding the limitations of QC applications in bioinformatics (Q4), several key issues have been identified. First, the initial phase of production-ready solutions in QC hardware—doing practical research is limited to a short amount of computational time/power which often strongly restricts the scope. Second, the state-of-the-art is in many QC-in-Bioinformatics related topics at the basic level and the application domain has not yet established strong foundations which cause many research paths to be closed. Third, there are no standards in terms of used datasets and metrics compared to the classic computation domain.

## Conclusions

Identifying key areas for future research at the intersection of QC and bioinformatics is crucial. The main direction should be to gain better foundations of underlying methods and go further with reproducible approaches on larger datasets. There should also be a greater contribution in creating standard metrics and methodologies to compare results in the quantum domain.

In the end, it should be noted that there is a bias towards successful research—if the results do not support the initial thesis, there is a risk that it will not be published. On one hand, failed research should be published only when it fulfils the non-obviousness criterion, but this causes an adverse bias—research showing no obvious failure of a given method is treated with reserve compared to successful ones.

**Key Points**

- This systematic review maps the methods and algorithms of quantum computing in bioinformatics, providing an overview of current trends and future research directions.
- The study addresses research questions concerning the current state, potential applications, challenges, and future prospects of quantum computing in bioinformatics.
- Quantum computing is becoming more popular in the bioinformatics community. However, both QC and its combination with bioinformatics are still in the early stages of development.

## Figures and Tables

**Figure A1:**
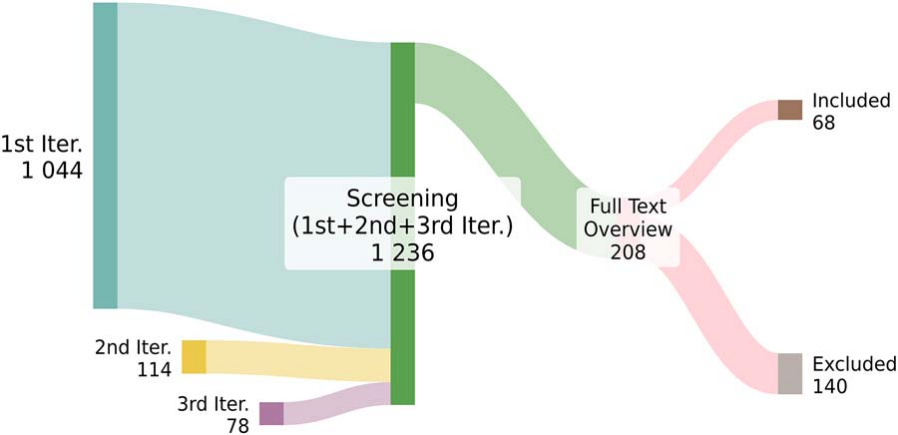
The diagram illustrates the flow of studies through the different stages of the selection process. The numbers represent the number of studies at each stage.

## Data Availability

The data underlying this article are available in the article.
